# Multi-Dimensional Characterization of Tetraspanins Reveals TSPAN6 as a Candidate Prognostic Hub in Kidney Renal Clear Cell Carcinoma

**DOI:** 10.3390/biology15201820

**Published:** 2026-10-12

**Authors:** Hajed Obaid A. Alharbi, Tarique Sarwar, Arshad Husain Rahmani

**Affiliations:** Department of Medical Laboratories, College of Applied Medical Sciences, Qassim University, Buraydah 51452, Saudi Arabiat.sarwar@qu.edu.sa (T.S.)

**Keywords:** KIRC, prognosis, TSPAN6, prognostic biomarker, PPIN, FFL

## Abstract

Kidney renal clear cell carcinoma (KIRC) is a major subtype of renal cell carcinoma with substantial molecular heterogeneity and variable clinical outcomes. Tetraspanins (TSPANs) are membrane proteins involved in cell adhesion, signaling, migration, tumor progression, and interactions with the tumor microenvironment (TME); however, their collective significance in KIRC remains insufficiently characterized. The expression profile of the TSPAN gene family was evaluated in the TCGA-KIRC cohort using GEPIA2. Differentially expressed TSPANs were further investigated through protein–protein interaction network (PPIN) and univariate survival analyses. Prognostically significant TSPANs were assessed for genomic alterations, promoter methylation, and associations with tumor-infiltrating immune cells. Feed-forward-loop (FFL) was constructed and visualized using Cytoscape. Thereafter, the findings were evaluated in an independent KIRC dataset. Several TSPANs displayed significant differential expression between KIRC and normal renal tissues. *TSPAN1* and *TSPAN6* were significantly associated with overall survival (OS), with higher expression corresponding to more favorable outcomes. Notably, higher *TSPAN6* expression was associated with improved OS but a higher estimated risk of relapse indicating an endpoint-dependent and potentially complex prognostic relationship. Promoter methylation of *TSPAN1* and *TSPAN6* showed associations with selected clinicopathological characteristics, while both genes demonstrated significant relationships with specific immune-cell populations. Network analysis identified a ZNF260–miR-6794-5p–*TSPAN6* FFL subnetwork motif, prioritizing *TSPAN6* as the hub TSPAN. *TSPAN6* was additionally detected among differentially expressed genes (DEGs) in an independent GEO cohort. This study identifies *TSPAN6* as a promising candidate biomarker in KIRC and provides a multi-dimensional framework linking TSPAN dysregulation with prognosis, epigenetic regulation, immune infiltration, and regulatory networks. Experimental and clinical validation is warranted.

## Simple Summary

Kidney renal clear cell carcinoma (KIRC) is the most common renal cell carcinoma (RCC) for which reliable molecular biomarkers are still needed. In this study, we systematically investigated the tetraspanin (TSPAN) gene family using multiomics and network-based analyses. Several TSPANs showed altered expression in KIRC, while *TSPAN1* and *TSPAN6* were significantly associated with overall and relapse-free survival. *TSPAN6* was further highlighted because of its strong relationship with promoter methylation, genomic alterations, immune-cell infiltration, and most importantly, a predicted ZNF260–miR-6794-5p–TSPAN6 regulatory network. Its differential expression was also observed in an independent dataset. These findings identify *TSPAN6* as a candidate prognostic biomarker and regulatory hub, although experimental and clinical validation is still required.

## 1. Introduction

RCC is the most common malignancy originating from the renal parenchyma and comprises a heterogeneous group of histological subtypes, of which KIRC is the predominant form. Kidney cancer represents a considerable global health burden, with approximately 434,840 new cases and 155,953 deaths estimated worldwide in 2022 [1]. The incidence of RCC has increased in several regions, reflecting changes in population demographics, lifestyle-associated risk factors, improved imaging and increased detection of incidental renal masses [2]. The etiology of RCC is multifactorial, involving genetic susceptibility, environmental exposure and metabolic factors. Established risk factors include cigarette smoking, obesity, hypertension, advancing age and family history, while hereditary syndromes such as VHL disease contribute to a smaller proportion of cases [2,3]. Clinical outcome is strongly influenced by tumor stage, histological characteristics and metastatic status. Patients with localized RCC generally have a favorable prognosis following surgical treatment, whereas metastatic disease is associated with substantially poorer survival [4]. Current management of RCC includes active surveillance or nephron-sparing surgery for selected localized tumors, radical nephrectomy for appropriate advanced localized disease, and systemic therapies for unresectable or metastatic disease. The therapeutic landscape of advanced RCC has evolved considerably with the introduction of VEGF-directed therapies, mammalian target of mTOR inhibitors and, more recently, ICIs targeting the PCD protein-1 (PD-1)/PD-L1 and CTLA-4 pathways [5]. Despite these advances, tumor recurrence, metastatic progression, treatment resistance and considerable molecular heterogeneity remain major clinical challenges. Therefore, identification of molecular determinants that can improve prognostic stratification and provide insights into the biological mechanisms underlying KIRC progression remains an important research priority.

TSPANs are a conserved family of 33 four-transmembrane proteins belonging to the TSPAN superfamily and are characterized by four transmembrane domains, small intracellular and extracellular loops, and a large extracellular loop containing conserved structural features [6,7]. Rather than functioning solely as conventional receptors, TSPANs organize specialized membrane microdomains known as TEMs through lateral interactions with integrins, GFRs, adhesion molecules and other signaling proteins [6,8]. Through these interactions, TSPANs regulate diverse CPs, including cell adhesion, migration, proliferation, differentiation, membrane trafficking, signal transduction and intercellular communication [6,7,8,9]. Increasing evidence has established that dysregulated TSPAN expression contributes to multiple stages of cancer development, including tumor-cell survival, EMT, invasion, metastatic dissemination, extracellular-vesicle biology and therapeutic resistance [9,10,11]. Importantly, TSPANs exhibit highly context-dependent functions; whereas some family members promote malignant progression, others can exert tumor-suppressive effects depending on the tumor type and cellular environment [9,12]. Their involvement in CSS and their accessibility at the plasma membrane have consequently generated considerable interest in their potential utility as diagnostic, prognostic and therapeutic biomarkers. However, despite the expanding literature on TSPANs in cancer, the collective contribution of the TSPAN family to KIRC remains incompletely characterized.

Previous studies have provided evidence that individual TSPAN members may have clinical and biological relevance in renal malignancies. Subsequent studies in other malignancies have further demonstrated that TSPAN members can regulate cancer-associated phenotypes through diverse molecular mechanisms. For example, *TSPAN1* has been implicated in EMT and metastasis through PI3K/AKT signaling, while *TSPAN7* has been associated with migration, proliferation and EMT in other tumor contexts [13,14]. More recently, Lu et al. performed a comprehensive investigation of *TSPAN7* in kidney neoplasms and reported that elevated *TSPAN7* expression was associated with poorer OS and RFS in a cohort of 370 patients. Their study further explored *TSPAN7* in relation to tumor purity, genomic alterations, immune infiltration, regulatory RNA networks, drug sensitivity and signaling pathways [15]. These findings collectively support the potential importance of TSPAN proteins in renal tumor biology; however, most previous investigations have focused on individual TSPAN members, particularly *TSPAN7*, rather than systematically examining the complete TSPAN family within KIRC.

Based on these observations, the present study aims to systematically characterize the TSPAN family in KIRC and identify TSPAN members with potential prognostic and biological significance. The study integrates differential-expression profiling, PPIN analysis, survival assessment, genomic alteration analysis, promoter-methylation profiling, tumor-immune infiltration analysis, FFL construction and independent GEO-based validation. This multi-dimensional strategy represents a major strength of the work because it moves beyond simple expression-based biomarker identification and examines potential relationships among TSPAN dysregulation, clinical outcome, epigenetic regulation, immune contexture and post-transcriptional regulatory mechanisms. The identification of *TSPAN6* as a hub member provides a specific candidate for subsequent mechanistic investigation and may contribute to the development of improved prognostic biomarkers for KIRC. Nevertheless, the current findings are predominantly derived from publicly available datasets and computational analyses and therefore establish associations rather than causality. Experimental validation at the RNA and protein levels, functional investigation using KIRC cell and animal models, and prospective clinical validation will be necessary to determine the biological and translational significance of *TSPAN6*. Future studies should also investigate whether *TSPAN6* influences tumor proliferation, migration, invasion, immune interactions and therapeutic response and whether modulation of its regulatory network can provide a basis for novel therapeutic strategies. Thus, this study provides a foundation for further experimental exploration of the *TSPAN6*-centered regulatory landscape in KIRC, while recognizing the need for functional and clinical validation before translation into routine biomarker or therapeutic applications.

## 2. Materials and Methods

### 2.1. Expression Analysis of TSPAN Family Across KIRC Cohort via GEPIA2

The HGNC database [16,17] (https://www.genenames.org/, accessed on 1 July 2026) was initially consulted to retrieve the complete list of officially approved protein-coding genes belonging to the TSPAN gene family. To assess the differential mRNA expression patterns of TSPAN gene family across TCGA-KIRC cohort matched with TCGA normal data, GEPIA2 web server [18] (http://gepia2.cancer-pku.cn/, accessed on 4 July 2026) was queried thereafter. Only those genes which were differentially expressed corresponding to a statistical threshold of p-value<0.05 and $\left|{log}_{2}(fold\;change)\right|>1$ were retained for further downstream analysis.

### 2.2. PPIN and Univariate Survival Analyses

We inputted all the significant differentially expressed TSPANs into STRING v12.0 database [19] (https://string-db.org/, accessed on 7 July 2026) to establish PPINs corresponding to medium confidence (interaction score>0.4) as well as low confidence (interaction score>0.15). Both of these PPINs were visualized thereafter via Cytoscape v3.10.4 [20]. The prognostic relevance of differentially expressed TSPANs was evaluated utilizing the KM plotter database [21,22] (https://kmplot.com/analysis/, accessed on 8 July 2026). The analysis was performed using the TCGA-KIRC cohort to investigate the associations of gene expression with OS and RFS. For every differentially expressed TSPAN, patients were stratified into high-and low-expression groups using the platform’s “Auto select best cutoff” function, which evaluates expression thresholds within the predefined range to identify the most informative separation between survival groups. KM survival curves were subsequently generated, and differences in survival distributions between the expression groups were assessed using the log-rank test. A log-rank p-value<0.05 was considered indicative of a statistically significant association between gene expression and patient prognosis.

### 2.3. Mutational and Promoter Methylation Analyses

All the prognostically significant TSPANs were entered into the cBioPortal web-based tool [23,24] (https://www.cbioportal.org/, accessed on 10 July 2026) in order to examine the mutations and putative CNAs. We selected the KIRC (TCGA, Firehose Legacy) and considered all samples for performing the mutational analysis. Next, we performed promoter methylation level analysis of prognostically significant TSPANs based on cancer stages, tumor grade, and nodal metastasis status by accessing UALCAN web-based tool [25,26] (http://ualcan.path.uab.edu/, accessed on 11 July 2026).

### 2.4. Tumor Infiltration Analysis

TIMER 3.0 [27] (http://timer.cistrome.org/, accessed on 14 July 2026) was utilized to assess the associations between prognostically significant TSPANs and the infiltration levels of neutrophils, NKTs, MPs, and monocytes across TCGA-KIRC cohort. Spearman’s correlation analysis was employed to determine the correlation coefficients, and a p-value<0.05 was considered indicative of statistical significance. Tumor purity was additionally assessed in relation to prognostically significant TSPANs expression.

### 2.5. 3-Node miRNA FFL Construction and Topological Analysis

ChEA3 database [28] (https://maayanlab.cloud/chea3/, accessed on 16 July 2026) was queried to capture statistically significant (i.e., p-value≤0.001) human TFs linked with prognostically significant TSPANs. Next, miRNA-mRNA along with miRNA-TF connections were captured from miRWalk v3.0 database [29] (http://mirwalk.umm.uni-heidelberg.de/, accessed on 16 July 2026) where only 3′UTR miRNAs having an interaction score>0.95 and a binding gap of 1 were considered. All these interaction pairs (i.e., miRNA-mRNA, miRNA-TF, and TF-mRNA) were amalgamated to build a 3-node miRNA FFL followed by visualization and topological analysis via Cytoscape. All FFL nodes were characterized using the following three complementary network-centrality measures: ND, betweenness centrality, and closeness centrality. The top 15 nodes based on each centrality were retained for comparative assessment. FFL motif(s) were prioritized based on their highest ranking across all these centrality measures.

### 2.6. External Validation via GEO

We queried NCBI-GEO [30] (https://www.ncbi.nlm.nih.gov/geo/, accessed on 22 July 2026) and entered keywords such as “KIRC” and “kidney renal cell carcinoma” in order to exhaustively search and retrieve KIRC-associated mRNA expression profile(s). Studies were considered for inclusion if they (i) contained samples derived from human patients along with appropriate healthy control samples, (ii) used an “expression profiling by array” experimental platform, and (iii) provided complete raw and processed expression data for the samples analyzed. Studies were excluded if they were case reports, conference abstracts, review articles, involved non-human experimental systems, lacked healthy control samples, or were based exclusively on cell-line experiments. The series matrix file corresponding to the selected GEO dataset was downloaded and subjected to QC to evaluate the integrity and suitability of the expression data for downstream analyses. Batch effects were subsequently addressed using the NOISeq v2.56.0 R package [31,32] to generate batch-adjusted expression profiles. The normalized expression values were then standardized by applying z-score transformation with the “scale” function. Probe-level identifiers were converted to official gene symbols according to the HGNC nomenclature using GEO2R. Where multiple probes corresponded to the same gene, their expression measurements were averaged to obtain a single representative value [33,34,35,36,37]. Genes exhibiting low variability, defined as the lowest 50% based on expression variation, were removed before downstream analysis [38]. Differential expression between the two experimental groups was assessed using an unpaired two-tailed *t*-test implemented through the limma v3.68.5 R package [39], from which gene-wise *p*-values and ${log}_{2}(fold\;change)$ estimates were obtained. DEGs were screened at a statistical cutoff of BH−p-value<0.05 and $\left|{log}_{2}(fold\;change)\right|>1$. Finally, the occurrence of prognostically significant TSPANs was checked in the corresponding DEG list obtained from GEO.

## 3. Results

### 3.1. Expression Analysis of TSPAN Family Across KIRC Cohort via GEPIA2

**Figure 1:**
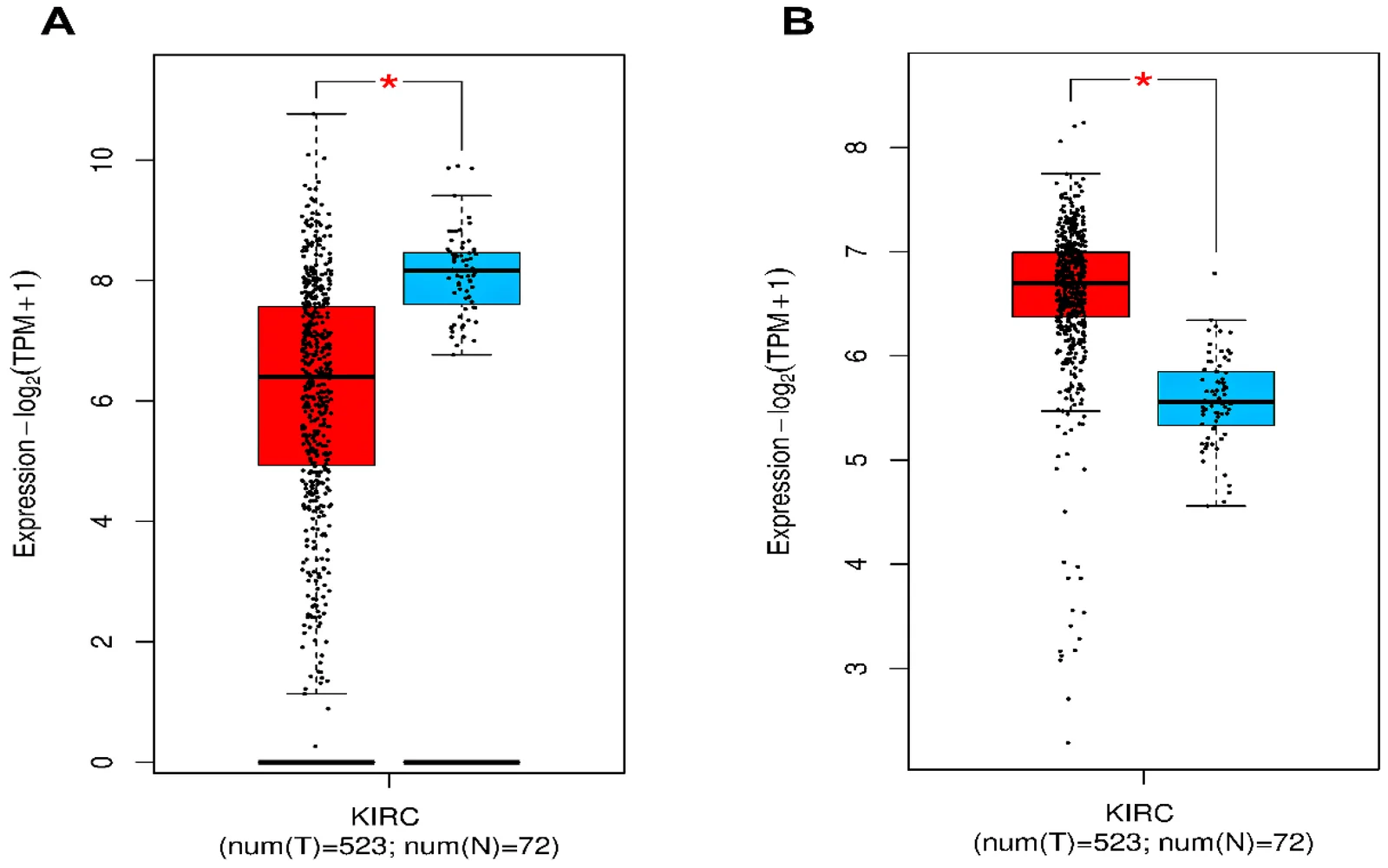

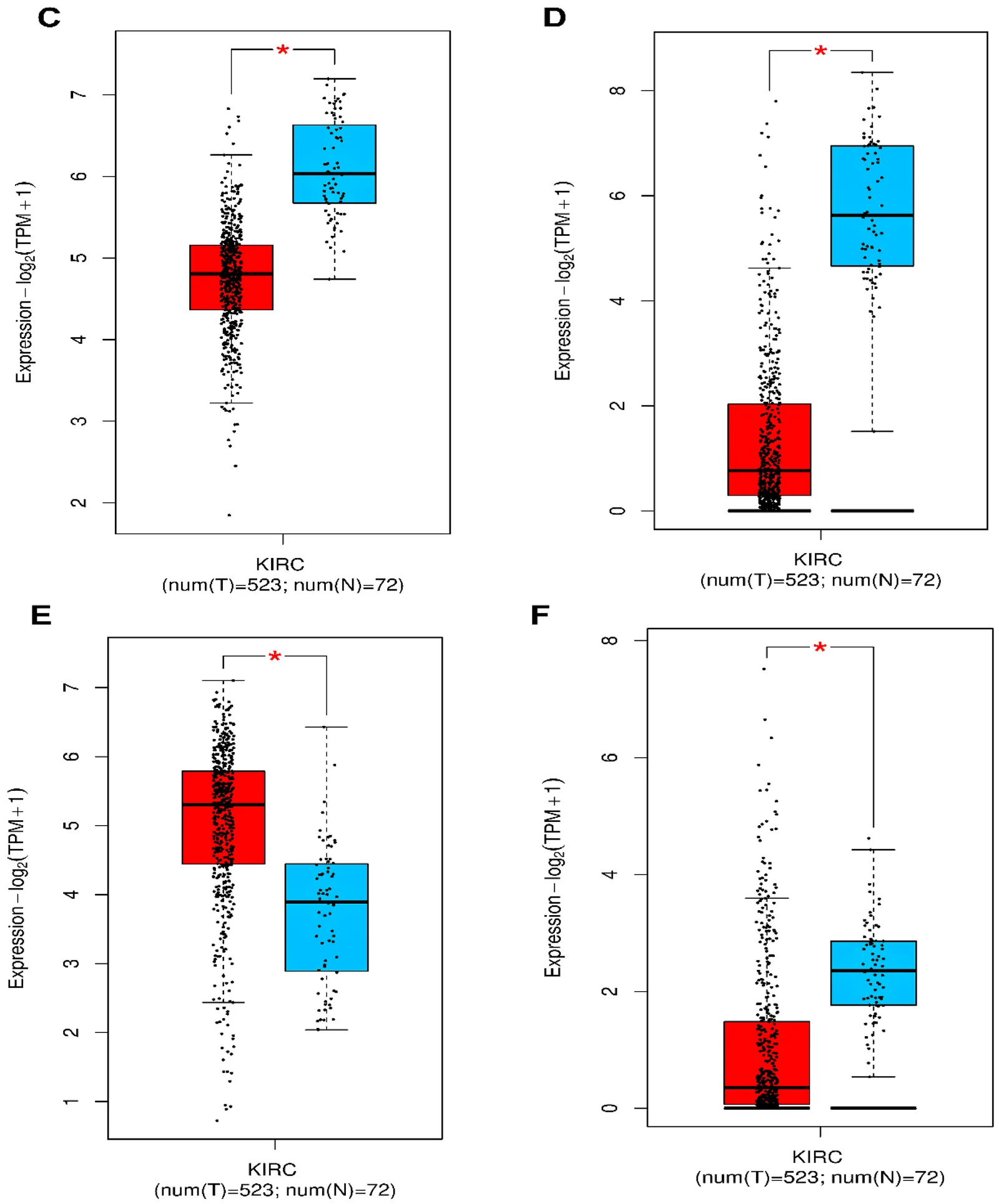

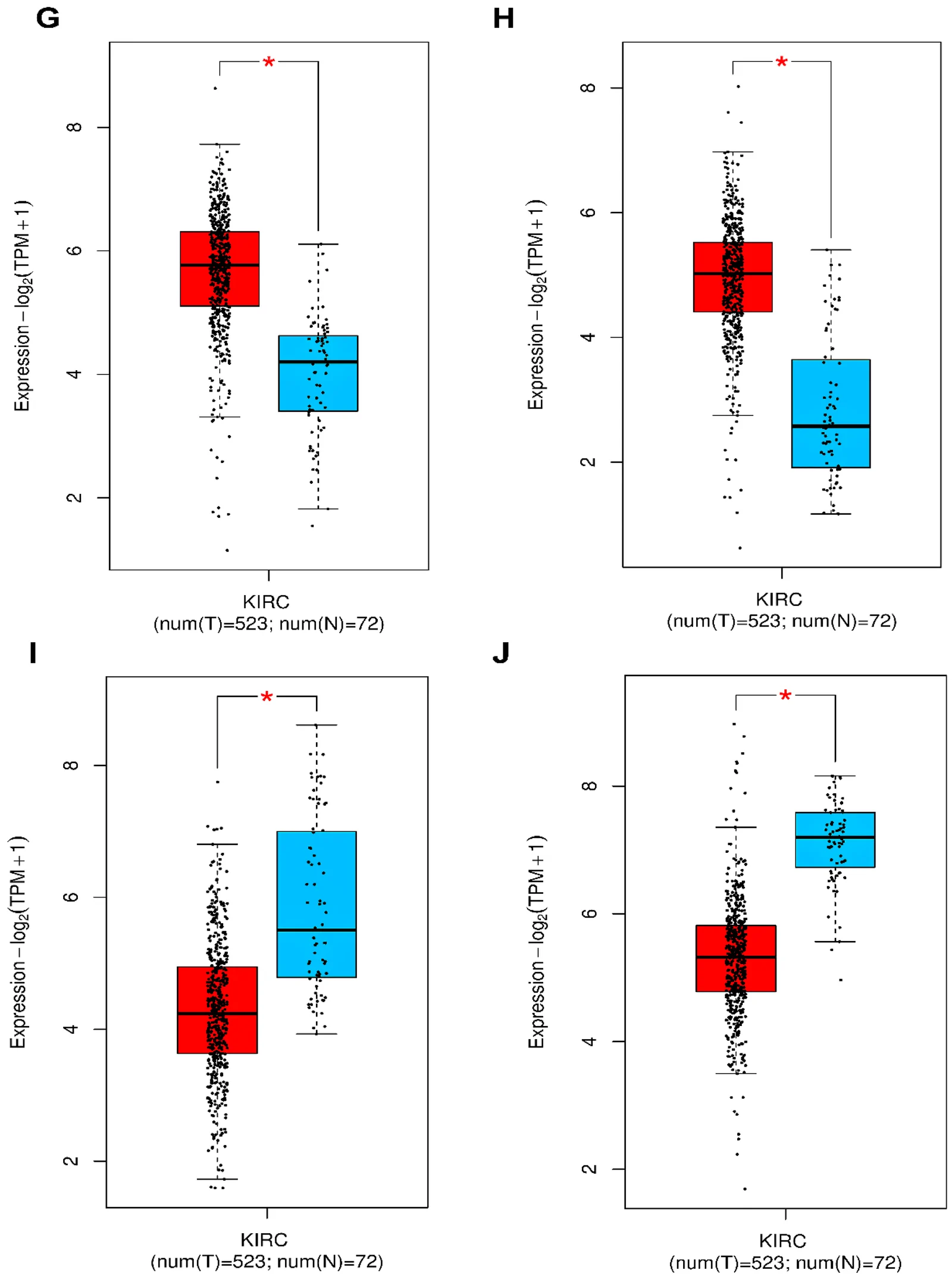
Box-and-whisker plots displaying the significant differential mRNA expression levels of (**A**) *TSPAN1*, (**B**) *TSPAN4*, (**C**) *TSPAN6*, (**D**) *TSPAN8*, (**E**) *TSPAN18*, (**F**) *TSPAN20*, (**G**) *TSPAN25*, (**H**) *TSPAN26*, (**I**) *TSPAN27*, (**J**) *TSPAN33* across 72 normal and 523 tumor patient samples. Red-and blue-colored shaded boxes indicate tumor and normal patient samples. * denotes significance at p-value<0.05 and $\left|{log}_{2}(fold\;change)\right|>1$.

Amongst all *TSPAN4*, *TSPAN18*, *TSPAN25*, and *TSPAN26* expression levels were significantly upregulated whereas *TSPAN1*, *TSPAN6*, *TSPAN8*, *TSPAN20*, *TSPAN27*, and *TSPAN33* expression levels were significantly downregulated in 523 tumor samples with respect to 72 normal samples as shown by the box-and-whisker plots in Figure 1.

### 3.2. PPIN and Univariate Survival Analyses

**Figure 2:**
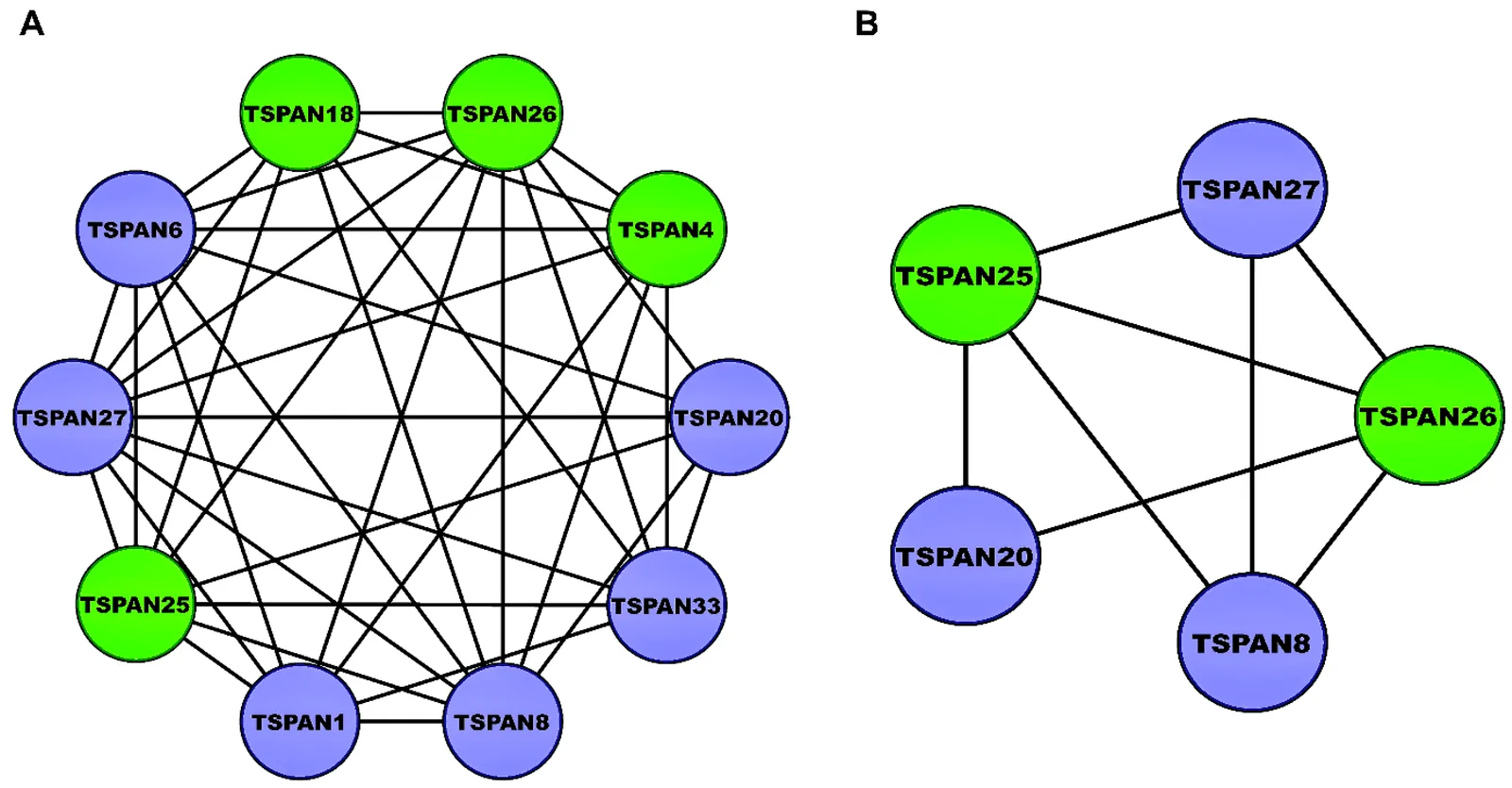
(**A**) Unweighted and undirected PPIN comprising 10 nodes and 38 edges corresponding to STRING interaction score > 0.15. (**B**) Unweighted and undirected PPIN comprising 05 nodes and 08 edges corresponding to STRING interaction score > 0.40. Green-and violet-colored nodes signify up-and downregulated expression status of DEGs.

**Figure 3:**
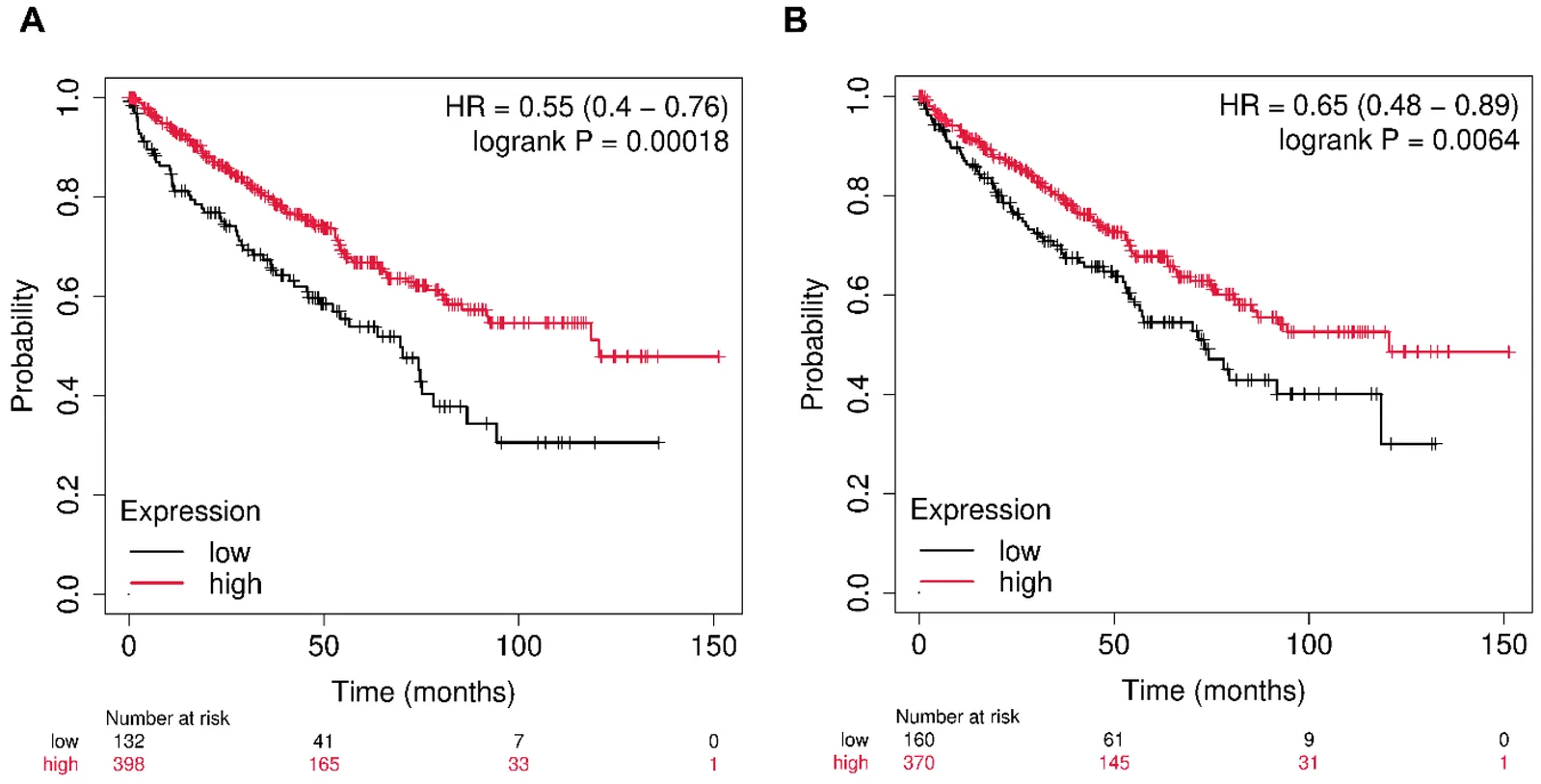

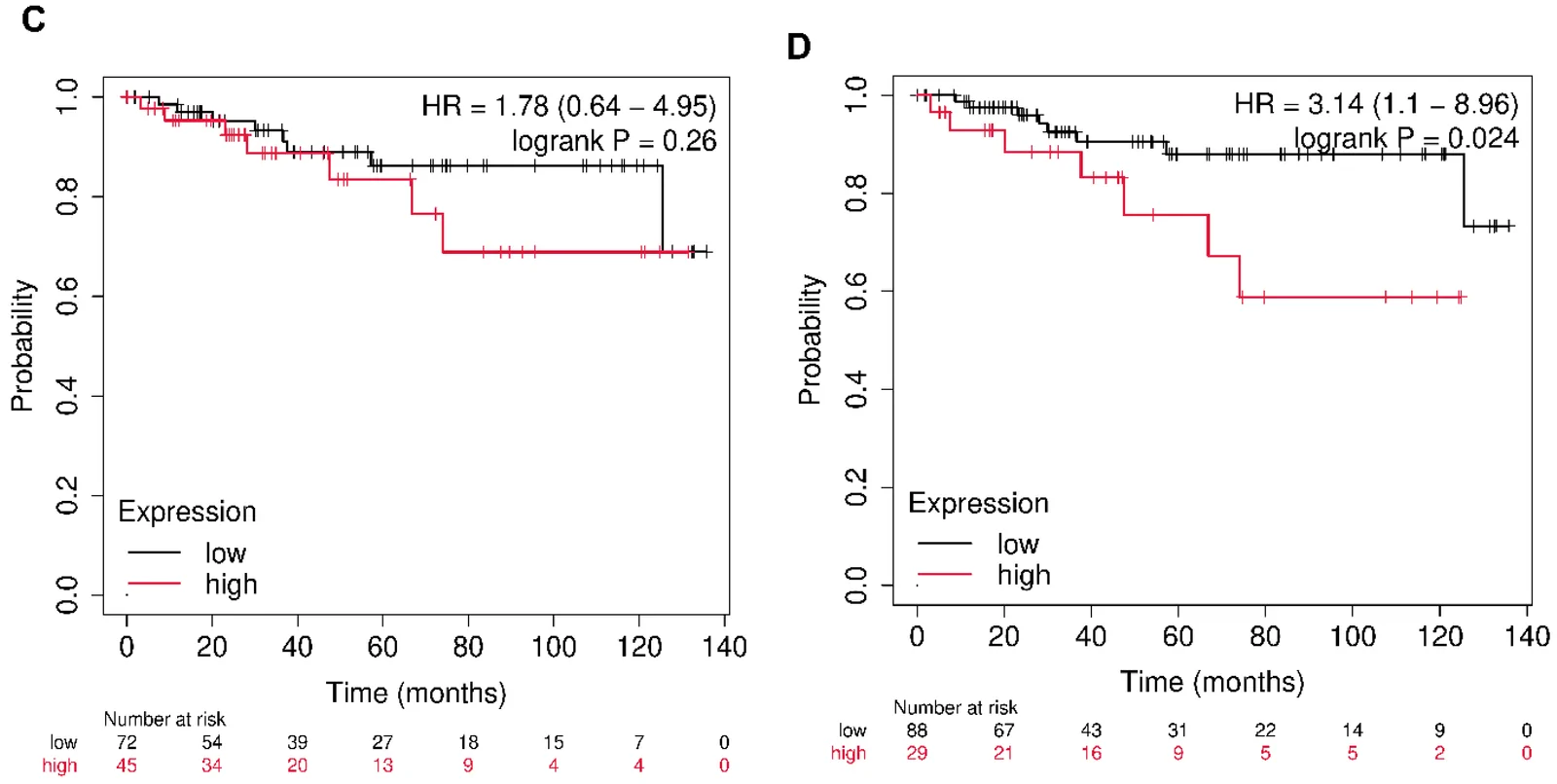
KM plots demonstrating OS of (**A**) *TSPAN1* and (**B**) *TSPAN6* across 530 TCGA-KIRC samples. KM plots demonstrating RFS of (**C**) *TSPAN1* and (**D**) *TSPAN6* across 530 TCGA-KIRC samples. Lines shaded in black and red resemble samples with reduced and greater expression levels, respectively.

We generated unweighted and undirected PPINs, as shown in Figure 2A,B, comprising 10 nodes joined by 38 edges for low-confidence setting in contrast to medium confidence setting comprising five nodes joined by eight edges, respectively. Patients with higher *TSPAN1* expression exhibited significantly better OS than those with lower expression (HR=0.55, log−rank P=0.00018), indicating that higher *TSPAN1* expression was associated with a reduced risk of death. Similarly, *TSPAN6* expression was significantly associated with favorable OS, with patients exhibiting higher *TSPAN6* expression showing better survival outcomes (HR=0.65, log−rank P=0.0064). These findings suggest that reduced expression of both *TSPAN1* and *TSPAN6* is associated with poorer OS in KIRC (Figure 3A,B). In contrast, the RFS analysis revealed differential associations for the two genes. *TSPAN1* expression was not significantly associated with RFS (HR=1.78, log−rank P=0.26). Conversely, *TSPAN6* expression showed a significant association with RFS (HR=3.14, log−rank P=0.024), with the high-expression group exhibiting a higher estimated risk of relapse (Figure 3C,D). Overall, KM Plotter analysis demonstrated that higher expression of both *TSPAN1* and *TSPAN6* was associated with favorable OS, whereas their associations with RFS differed, with no significant association observed for *TSPAN1* and an unfavorable RFS association observed for *TSPAN6*.

### 3.3. Mutational and Promoter Methylation Analyses

**Figure 4:**
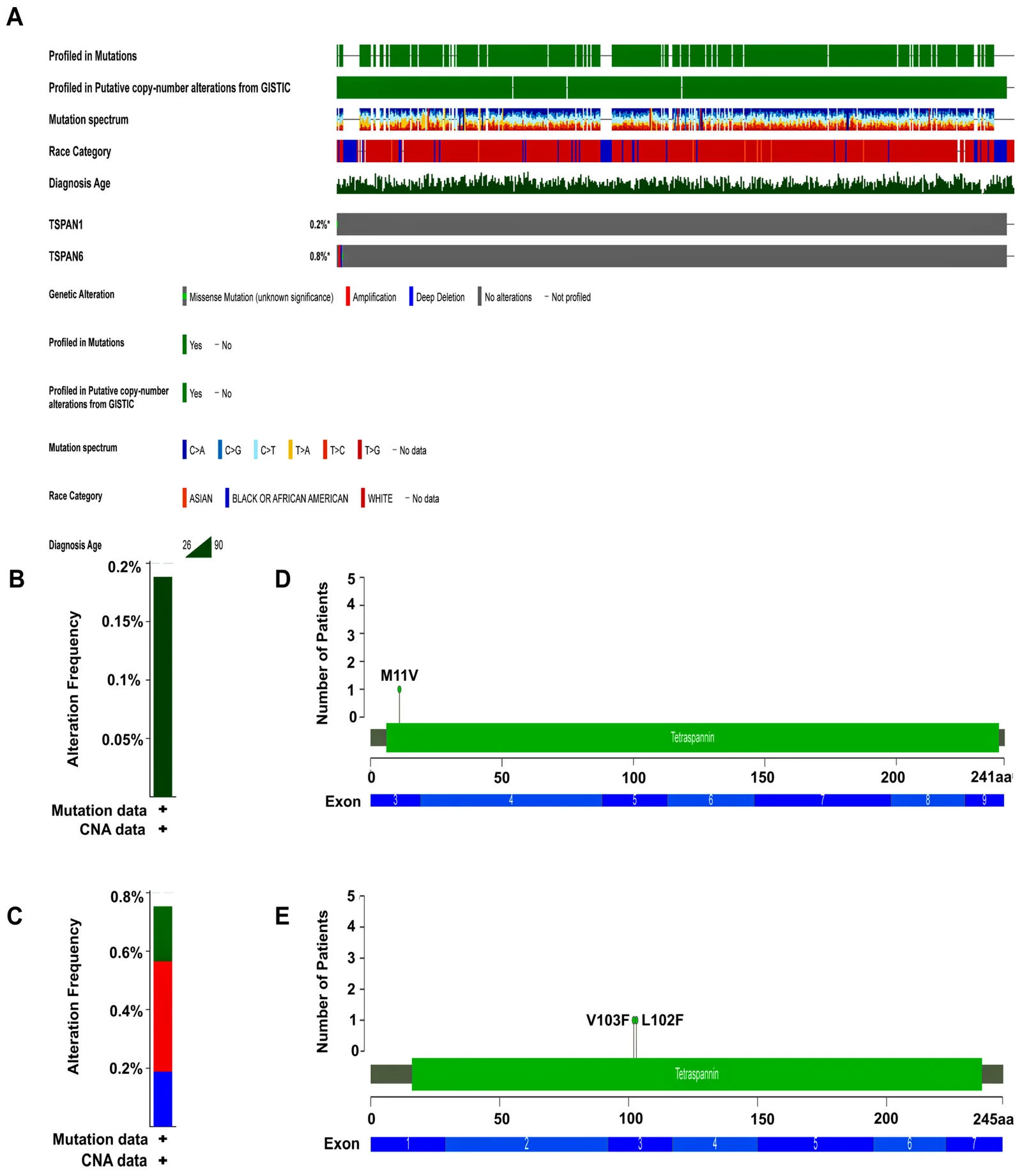
(**A**) OncoPrint offers a comprehensive snapshot of genomic alterations in *TSPAN1* and *TSPAN6* among 530 patients included in the TCGA-KIRC cohort. First, second, third, fourth, and fifth rows depicts the profiled in mutations, profiled in putative CNAs from GISTIC, mutation spectrum, race, diagnosis age. Bottom rows exhibit patient diagnosis age, race category, mutation spectrum, genetic alteration types (i.e., missense, amplification, deep deletion, no alterations, not specified). Barplots displaying the alteration frequencies of (**B**) *TSPAN1* and (**C**) *TSPAN6* across the 530 TCGA-KIRC patient samples. Here, green, blue, and red bars imply missense mutation, deep deletion, and amplification, respectively. Lollipop plots illustrating 0.2% somatic mutations each in protein domains of (**D**) *TSPAN1* and (**E**) *TSPAN6*. The gray horizontal bar indicates the full length of each protein, with the corresponding amino acid positions shown beneath it. Protein domains are highlighted using a green box, while mutation positions and their frequencies are represented by vertical lines ending in lollipop-shaped markers.

**Figure 5:**
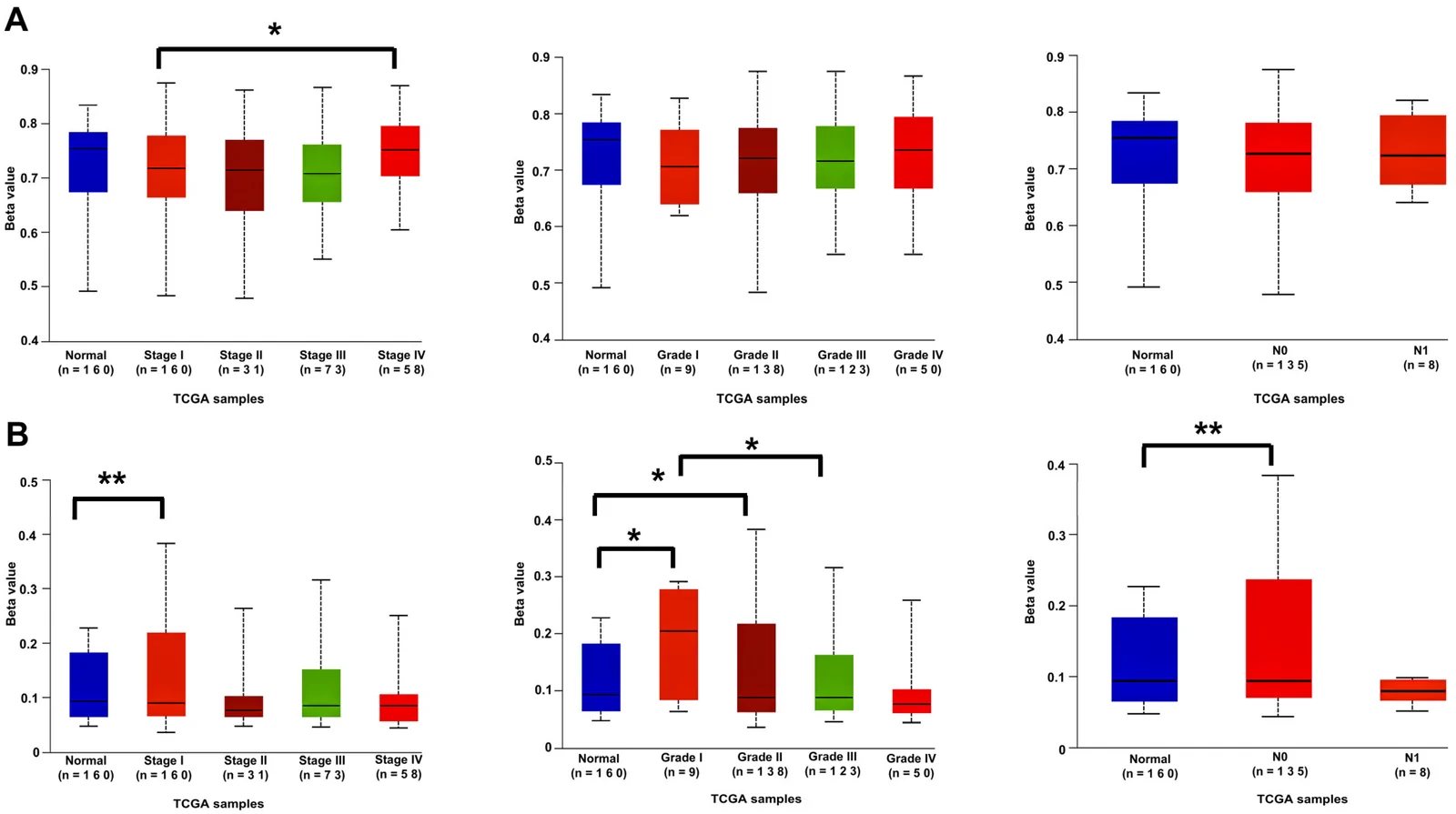
Box-and-whisker plots showing the distribution of promoter methylation according to normal tissue with respect to different tumor stages (stage I–IV), tumor grades (grade I–IV), and nodal metastasis status (N0 and N1) in case of (**A**) *TSPAN1* and (**B**) *TSPAN6*. * *p*-value < 0.05, ** *p*-value < 0.01. The beta value on the y-axis represents the degree of DNA methylation, ranging from 0 (completely unmethylated) to 1 (completely methylated). Beta values of 0.70–0.50 were considered indicative of hypermethylation, whereas values of 0.30–0.25 were considered indicative of hypomethylation.

Oncoprint summary as shown in Figure 4A revealed that *TSPAN1* and *TSPAN6* were both altered in <1% (5/530) patient samples individually. Amongst all patients, eight belonged to Asian race, 56 belonged to black race, and 466 belonged to white race. Also, the diagnosis age of patients varied from 26 to 90, with 96% patients aged ≥40 while 4% patients having age ≤40. Figure 4B,C represents overall alteration frequencies of *TSPAN1* and *TSPAN6* based on the cancer-type summary analysis. Missense mutation frequency of 0.19% (1/530) was solely reported in case of TSPAN1. Also, *TSPAN6* reported 0.19% (1/530) missense mutation, 0.38% (2/530) amplification, and 0.19% (1/530) deep deletion mutation frequencies, respectively. Lollipop plots as shown in Figure 4D,E display the frequency and location of all possible mutations in Pfam protein domains in case of *TSPAN1* and *TSPAN6*. Both of them exhibited 0.2% somatic mutation frequency individually with one missesnse mutation in case of *TSPAN1* while two there were missesnse mutations in case of *TSPAN6*. The box-and-whisker plots shown in Figure 5A,B illustrate the promoter methylation levels of *TSPAN1* and *TSPAN6* according to cancer stage, tumor grade, and nodal metastasis. For *TSPAN1*, promoter methylation analysis based on cancer stage revealed a significant increase in methylation between stage I and stage IV tumors. However, no significant differences were observed among the remaining cancer-stage comparisons. Similarly, *TSPAN1* promoter methylation did not differ significantly according to tumor grade or nodal metastasis status. In contrast, *TSPAN6* promoter methylation analysis demonstrated a significant difference between normal tissues and stage I tumors. With respect to tumor grade, significant differences were observed between normal and grade I, normal and grade II, and grade I and grade III tumors. Furthermore, analysis according to nodal metastasis revealed a significant difference between N0 and N1 tumors, with higher *TSPAN6* promoter methylation in the N0 group. Collectively, these findings indicate that promoter methylation of *TSPAN1* is primarily associated with the transition from stage I to stage IV, whereas *TSPAN6* methylation shows significant associations with selected tumor stages, grades, and nodal metastatic status.

### 3.4. Tumor Infiltration Analysis

**Figure 6:**
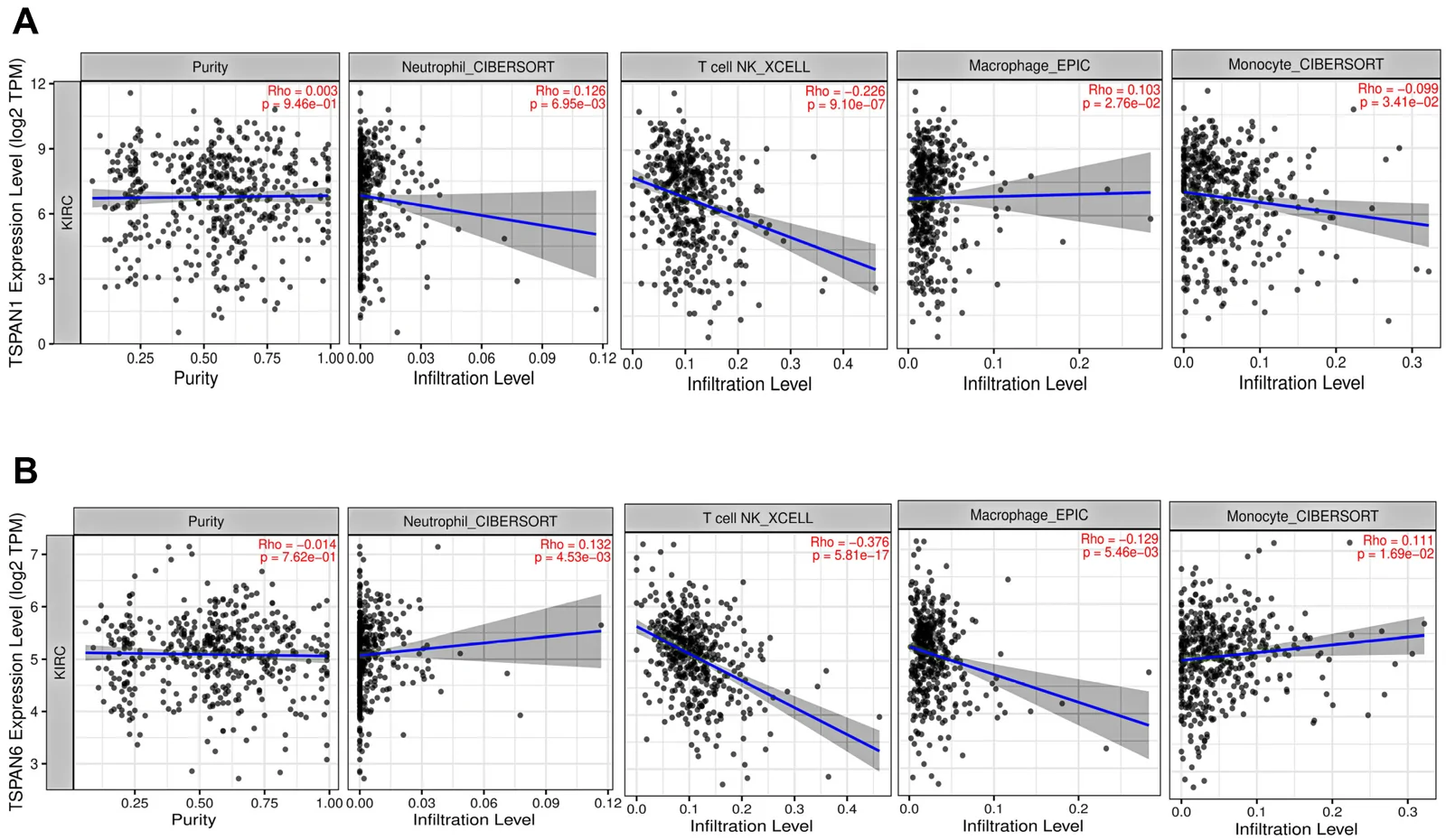
Scatterplots demonstrating significant associations between infiltrating levels of neutrophils, NKTs, MPs, monocytes with (**A**) *TSPAN1* and (**B**) *TSPAN6* across TCGA-KIRC cohort. Additionally, mRNA expression levels in relation to tumor purity are illustrated in the left panel. For each scatter plot, Spearman’s correlation coefficient and the associated *p*-value are provided.

*TSPAN1* mRNA expression levels revealed significant positive correlations with neutrophils (r=0.126, $p\text{-}value=6.95\times 10^{-3}$) and MPs (r=0.103, $p\text{-}value=2.76\times 10^{-2}$) in contrast to significant negative correlations with monocytes (r=−0.099, $p\text{-}value=3.41\times 10^{-2}$) and NKTs (r=−0.226, $p\text{-}value=3.41\times 10^{-2}$), as evidenced by scatterplots in Figure 6A. *TSPAN6* mRNA expression levels revealed significant negative correlations with MPs (r=−0.129, $p\text{-}value=5.46\times 10^{-3}$) and NKTs (r=−0.376, $p\text{-}value=5.81\times 10^{-17}$) in contrast to significant positive correlations with neutrophils (r=0.132, $p\text{-}value=4.53\times 10^{-3}$) and monocytes (r=0.111, $p\text{-}value=1.69\times 10^{-2}$), as evidenced by scatterplots in Figure 6B. Lastly, *TSPAN1* and *TSPAN6* revealed insignificant positive (r=0.003, $p\text{-}value=9.46\times 10^{-1}$) and negative (r=−0.014, $p\text{-}value=7.62\times 10^{-1}$) correlations with tumor purity across the TCGA-KIRC cohort.

### 3.5. Three-Node miRNA FFL Topological Analysis

Figure S1A shows the three-node miRNA FFL comprising 495 nodes and 1325 edges. Among the identified edges, TF–mRNA interactions accounted for 8 edges, whereas miRNA–TF and miRNA–mRNA interactions comprised 788 and 529 edges, respectively. The FFL consisted of 485 miRNAs, eight TFs, and two mRNAs. The degree of connectivity ranged from 2 to 275 for TFs, 2 to 6 for miRNAs, and 144 to 393 for mRNAs. Mean degree values for TFs, miRNAs, and mRNAs were 99.5, 2.71, and 268.5, respectively. Tables S1–S3 list top 15 FFL elements ranked on the basis of ND, betweenness, and closeness. Based on the highest rankings as per all these centrality measures, we chose the highest-order subnetwork FFL motif comprising one TF (*ZNF260*), one miRNA (miR-6794-5p), and one mRNA (*TSPAN6*), as shown in Figure S1B. Hence, we considered *TSPAN6* as our hub TSPAN member.

### 3.6. External Validation via GEO

We picked the GSE53757 dataset, which consists of 72 paired normal and adjacent tumor tissue samples obtained from patients with KIRC. Applying the aforementioned threshold criteria, a total of 3451 DEGs (1719 downregulated + 1732 upregulated) were identified (Table S4). Notably, *TSPAN6* was also identified among the DEGs in the independent GSE53757 KIRC cohort, providing external validation of the reproducibility of *TSPAN6* differential expression in an independent dataset.

## 4. Discussion

By integrating differential expression profiling, PPIN analysis, survival analysis, genomic and epigenetic characterization, immune-infiltration analysis, FFL construction and independent GEO validation, the present work extends beyond conventional single-gene expression analysis. This approach is biologically relevant because TSPANs function as organizers of membrane-associated molecular complexes and can influence cell adhesion, migration, receptor signaling, membrane trafficking and communication between tumor cells and their TME. Therefore, the heterogeneous expression profile observed among TSPAN family members in KIRC may reflect selective remodeling of membrane-associated signaling networks during renal tumor development rather than a uniform role of the entire TSPAN family. Our analysis identified differential expression of multiple TSPAN genes, with *TSPAN4*, *TSPAN18*, *TSPAN25* and *TSPAN26* being upregulated, whereas *TSPAN1*, *TSPAN6*, *TSPAN8*, *TSPAN20*, *TSPAN27* and *TSPAN33* were downregulated in KIRC compared with normal renal tissues. Such heterogeneity is consistent with the well-recognized context-dependent behavior of TSPANs in cancer, where individual family members may either promote or suppress tumor progression depending on the cellular and molecular environment.

Among the differentially expressed TSPANs, *TSPAN1* and *TSPAN6* demonstrated significant associations with OS, with higher expression of both genes corresponding to more favorable outcomes. The favorable prognostic association of *TSPAN1* in KIRC is notable because *TSPAN1* has been reported to promote EMT and metastasis through PI3K/AKT signaling in cholangiocarcinoma [13]. This difference reinforces the concept that the functional consequences of TSPAN expression are tumor-context dependent. A molecule that contributes to aggressive behavior in one malignancy may be associated with a less aggressive molecular phenotype in another. *TSPAN6* was prioritized as the more compelling candidate because it showed a significant association with both OS and RFS, although the direction of these associations was not identical. High *TSPAN6* expression was associated with improved OS, whereas its relationship with RFS suggested a higher estimated risk of relapse. This apparent discordance should not be overlooked, as OS and RFS capture different aspects of disease biology. A molecular feature may be associated with long-term survival while simultaneously reflecting biological characteristics related to recurrence, treatment response or post-treatment tumor persistence. Therefore, the present findings do not support the interpretation of *TSPAN6* as a universally protective factor in KIRC. Instead, they suggest that *TSPAN6* may participate in more complex BPs whose consequences differ according to the clinical endpoint being examined.

Nevertheless, the favorable OS association observed for *TSPAN6* has biological support from recent experimental studies in other cancer systems. Andrijes et al. demonstrated that *TSPAN6* acts as a regulator of colorectal carcinogenesis and that loss of *TSPAN6* enhances tumor development through increased EGFR-dependent signaling. Mechanistically, *TSPAN6* was shown to regulate the production of extracellular vesicles containing transmembrane TGF-α, thereby influencing activation of the EGFR signaling axis [40]. More recently, Humbert et al. provided further evidence that *TSPAN6* can suppress RAS-driven tumor progression [41]. In pancreatic cancer xenografts and genetically engineered LC models, loss of *TSPAN6*-enhanced tumor initiation and malignant progression, whereas increased *TSPAN6* expression reduced tumor growth and metastatic dissemination. Mechanistically, *TSPAN6* interacted with EGFR and suppressed EGFR-induced RAS activation and downstream ERK signaling. The same study also linked reduced *TSPAN6* expression with EMT-associated phenotypes and poor prognosis in selected epithelial cancers. These experimental observations provide a biologically plausible framework for the finding that higher *TSPAN6* expression is associated with improved OS in KIRC. However, because these mechanisms have not yet been experimentally demonstrated in KIRC, it would be inappropriate to conclude that *TSPAN6* suppresses KIRC progression through EGFR–RAS–ERK signaling. Instead, this pathway represents an important mechanistic hypothesis for future investigation.

The genomic alteration analysis provides an additional perspective on the potential mechanisms underlying *TSPAN1* and *TSPAN6* dysregulation in KIRC. Both genes exhibited somatic alterations, with missense mutations detected in *TSPAN1* and *TSPAN6*, while *TSPAN6* additionally showed amplification and deep-deletion events. The occurrence of both sequence-level and CNAs suggests that genomic variation may contribute to the molecular heterogeneity of these TSPANs in KIRC. For *TSPAN6*, CNAs are particularly relevant because amplification or deep deletion can alter gene dosage and potentially influence transcript and protein abundance. Given that TSPAN6 is a four-transmembrane protein involved in organizing TSPAN-enriched membrane microdomains, genomic alterations affecting its abundance or protein structure could potentially modify interactions with GFRs, integrins and other membrane-associated signaling molecules. This possibility is biologically relevant to the EGFR–RAS–ERK signaling axis, as experimental studies have demonstrated that *TSPAN6* can interact with EGFR and restrain EGFR-induced RAS activation and downstream ERK signaling, whereas loss of *TSPAN6* enhances RAS-driven tumor progression [41]. Therefore, genomic loss or functionally disruptive alterations in *TSPAN6* could potentially contribute to a *TSPAN6*-low state associated with more aggressive tumor biology. However, the present study does not establish a direct relationship between individual *TSPAN6* alterations and its expression, signaling activity or patient outcome. Moreover, the coexistence of amplification and deep deletion suggests genomic heterogeneity among KIRC tumors rather than a uniform alteration pattern.

The promoter-methylation analysis provides an additional dimension to the potential regulation of *TSPAN1* and *TSPAN6* in KIRC. *TSPAN1* methylation was significantly different between stage I and stage IV tumors, whereas *TSPAN6* methylation showed associations with selected tumor stages, grades and nodal-metastatic status. The association of *TSPAN6* methylation with tumor grade and LN involvement is particularly interesting because these clinicopathological characteristics are related to disease aggressiveness. One possible interpretation is that epigenetic alterations may contribute to the dysregulated expression of *TSPAN6* during KIRC development or progression. The immune-infiltration findings further suggest that *TSPAN1* and *TSPAN6* may be associated with differences in the KIRC TME. *TSPAN1* expression was positively associated with neutrophils and MPs and negatively associated with monocytes and NKT cells, whereas *TSPAN6* showed positive associations with neutrophils and monocytes and negative associations with MPs and NKT cells. These observations are biologically relevant because immune-cell composition can substantially influence tumor progression, immune surveillance and therapeutic response. However, the current findings should be interpreted as transcriptomic associations rather than evidence that *TSPAN6* directly recruits or regulates these immune populations. TSPANs are known to participate broadly in cell–cell interactions and immune processes, making such associations biologically plausible. In addition, emerging work on *TSPAN6* in other tumor systems suggests that this molecule may participate in TME interactions, although these relationships remain highly context-dependent. Therefore, the distinct immune correlation profile observed for *TSPAN6* in KIRC warrants further investigation using single-cell transcriptomic approaches, spatial analysis and experimental co-culture models.

The regulatory-network analysis provided an additional rationale for prioritizing *TSPAN6*. Centrality-based analysis identified a ZNF260–miR-6794-5p–*TSPAN6* FFL subnetwork motif, positioning *TSPAN6* within a potentially coordinated transcriptional and post-transcriptional regulatory circuit. FFLs are important network motifs that can modulate the timing, robustness and context specificity of gene expression. The identification of *TSPAN6* within the highest-ranking motif therefore suggests that its dysregulation in KIRC may not occur independently but could be influenced by coordinated regulatory mechanisms involving both a TF and a miRNA. This observation is particularly relevant because altered regulatory networks are increasingly recognized as important contributors to cancer-associated gene-expression programs. Nevertheless, the ZNF260–miR-6794-5p–*TSPAN6* interaction identified here is prediction-based and requires experimental validation. ZNF260 is a zinc-finger TF and is reported as a potential prognostic marker in KIRC, with its expression showing a favorable survival association in KIRC, supporting the possibility that ZNF260 may participate in KIRC-associated transcriptional regulation. In contrast, emerging evidence provides a more direct cancer-related role for miR-6794-5p. Choi et al. demonstrated that tumor-derived miR-6794-5p promotes cancer aggressiveness by suppressing *SOCS1* and activating the JAK1/STAT3 pathway, while extracellular-vesicle-associated miR-6794-5p was transferred to MPs and induced M2 polarization, resulting in an immunosuppressive TME [42]. Thus, the predicted ZNF260–miR-6794-5p–TSPAN6 axis may represent a potential interface between transcriptional regulation, post-transcriptional control and tumor–immune interactions.

Multiple-testing considerations represent an additional limitation of the present study. To address the potential for false-positive findings in the high-dimensional GEO DEA, the BH-FDR adjustment of the gene-wise *p*-values was incorporated [43]. Thus, the identification of DEGs in the independent GEO cohort was based on FDR-adjusted significance rather than nominal *p*-values alone. Nevertheless, some of the other analyses performed in this study involved multiple comparisons within biologically predefined gene families, immune-cell populations, or candidate regulatory relationships and were conducted using the statistical frameworks provided by the respective analytical platforms. Therefore, although FDR correction substantially reduces the risk of false-positive findings in the genome-wide GEO analysis, residual multiplicity-related considerations cannot be completely excluded across the integrated analytical workflow. The findings should consequently be interpreted within the context of the exploratory and integrative nature of the study and should be further evaluated through independent cohorts and experimental validation.

## 5. Conclusions

This study provides a multidimensional characterization of TSPAN gene dysregulation in KIRC. Several TSPANs showed significant differential expression between KIRC and normal renal tissues, highlighting their heterogeneous involvement in renal tumor biology. Among the prognostically significant members, *TSPAN1* and *TSPAN6* were associated with OS. Importantly, higher *TSPAN6* expression was associated with improved OS but with a higher estimated risk of relapse based on RFS analysis, highlighting an endpoint-dependent and potentially complex prognostic relationship. *TSPAN6* was further prioritized as a hub TSPAN based on its position within the ZNF260–miR-6794-5p–TSPAN6 FFL. Its association with RFS suggests a potentially complex relationship with disease recurrence. *TSPAN6* also demonstrated genomic and promoter-methylation alterations associated with selected clinicopathological characteristics. Its significant relationships with specific immune-cell populations further suggest a possible connection with the KIRC TME. The identification of *TSPAN6* as a differentially expressed gene in an independent GEO cohort provides additional support for the reproducibility of this finding. Collectively, our findings identify *TSPAN6* as a candidate prognostic biomarker and potential regulatory hub in KIRC. Its consistent association with improved OS and RFS supports its potential prognostic relevance; however, independent clinical validation and multivariable analysis in adequately annotated cohorts are required to establish its prognostic value.

## Data Availability

Microarray mRNA dataset used in our study was downloaded from NCBI-GEO available at https://www.ncbi.nlm.nih.gov/geo/query/acc.cgi?acc=GSE53757, accessed on 22 July 2026.

## Supplementary Materials

The following supporting information can be downloaded at https://www.mdpi.com/article/10.3390/biology15201820/s1, Figure S1: (A) Unweighted and undirected miRNA FFL comprising 495 nodes and 1325 edges. (B) Highest-order subnetwork FFL motif comprising one TF (ZNF260), one miRNA (miR-6794-5p), and one mRNA (TSPAN6). Cyan-colored rectangular nodes signify miRNAs, orange-colored diamond nodes signify TFs, and blue-colored circular nodes signify downregulated mRNA, respectively.; Table S1: List of top 10 FFL elements ranked on the basis of node degree; Table S2: List of top 10 FFL elements ranked on the basis of betweenness; Table S3: List of top 10 FFL elements ranked on the basis of Closeness; Table S4: List of DEGs in paired KIRC samples.

## Abbreviations

LC, lung cancer; KIRC, kidney renal clear cell carcinoma; TSPAN, tetraspanin; PPIN, protein–protein interaction network; BP, biological process; FFL, feed-forward-loop; GEO, gene expression omnibus; NCBI, National Center for Biotechnology Information; OS, overall survival; RFS, relapse-free survival; KM, Kaplan–Meier; DEG, differentially expressed gene; RCC, renal cell carcinoma; VHL, von Hippel–Lindau; VEGF, vascular endothelial growth factor; mTOR, rapamycin; ICI, immune-checkpoint inhibitor; PCD, programmed cell death; CTLA-4, cytotoxic T-lymphocyte-associated protein 4; TEM, tetraspanin-enriched microdomain; GFR, growth-factor receptor; EMT, epithelial–mesenchymal transition; CP, cellular process; CSS, cell-surface signaling; RNA, ribonucleic acid; HGNC, HUGO Gene Nomenclature Committee; GEPIA, Gene Expression Profiling Interactive Analysis; STRING, Search Tool for the Retrieval of Interacting Genes/Proteins; TCGA, The Cancer Genome Atlas; MP, macrophage; NKT, natural killer T; TF, transcription factor; miRNA, microRNA; mRNA, messenger RNA; UTR, untranslated region; BC, batch correction; CNA, copy-number alteration; HR, hazard ratio; CI, confidence interval; TME, tumor microenvironment; LN, lymph-node; BH, Benjamini–Hochberg; QC, quality check; ND, node degree; FDR, false discovery rate.
