# Supplementary material for: Multi-Dimensional Characterization of Tetraspanins Reveals TSPAN6 as a Candidate Prognostic Hub in Kidney Renal Clear Cell Carcinoma
Source: Biology (Basel). 2026 Oct 12;15(20):1820. doi: 10.3390/biology15201820 (PMC13701831; doi:10.3390/biology15201820)
Supplement: Supplementary file 1 [file biology-15-01820-s001.zip › Figure S1.pdf]

<u>Supplementary Data</u>

Multi-Dimensional Characterization of Tetraspanins Reveals TSPAN6 as a Candidate Prognostic Hub in Kidney renal clear cell carcinoma

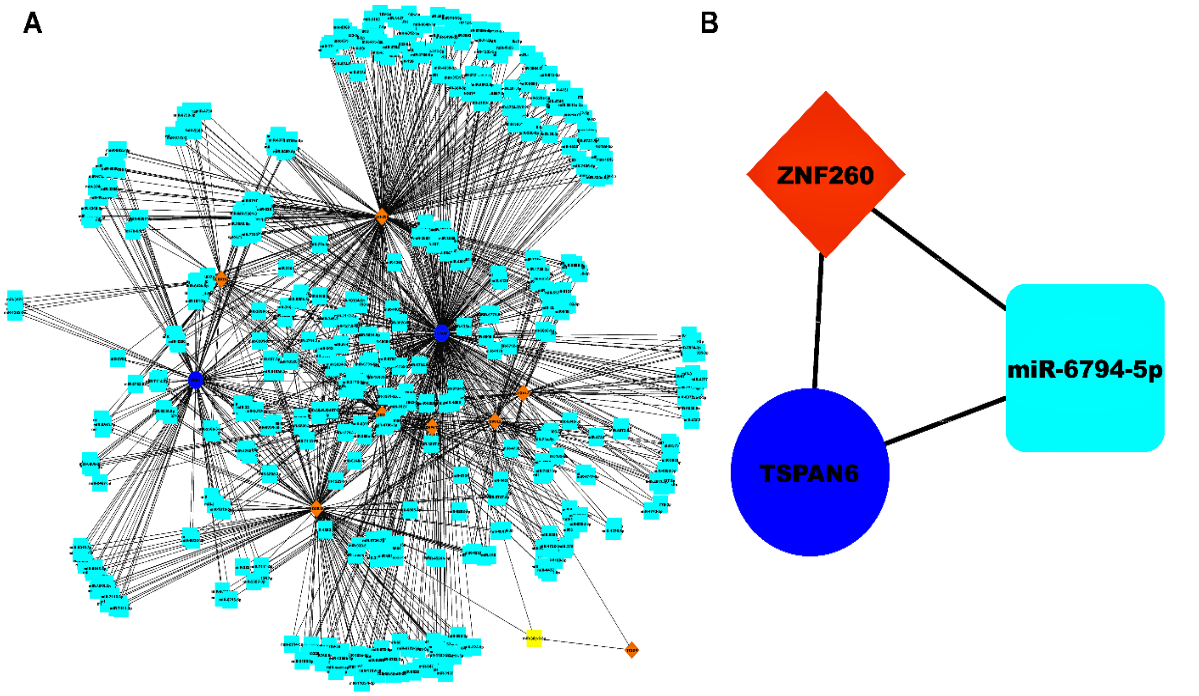

Figure S1. ***(A)*** *Unweighted and undirected miRNA FFL comprising 495 nodes and 1325 edges.* ***(B)*** *Highest-order subnetwork FFL motif comprising one TF (ZNF260), one miRNA (miR-6794-5p), and one mRNA (TSPAN6). Cyan-colored rectangular nodes signify miRNAs, orange-colored diamond nodes signify TFs, and blue-colored circular nodes signify downregulated mRNA, respectively.*
